# Mitogen-activated protein kinase 4-like carrying an MEY motif instead of a TXY motif is involved in ozone tolerance and regulation of stomatal closure in tobacco

**DOI:** 10.1093/jxb/erw173

**Published:** 2016-04-28

**Authors:** Yuki Yanagawa, Hiroshi Yoda, Kohei Osaki, Yuta Amano, Mitsuko Aono, Shigemi Seo, Kazuyuki Kuchitsu, Ichiro Mitsuhara

**Affiliations:** ^1^Institute of Agrobiological Sciences, NARO, 2-1-2 Kannondai, Tsukuba, Ibaraki 305–8602, Japan; ^2^Department of Applied Biological Science, Tokyo University of Science, Noda, Chiba 278–8510, Japan; ^3^Environmental Biology Division, National Institute for Environmental Studies, Tsukuba 305–8506, Japan; ^4^Imaging Frontier Center, Tokyo University of Science, Noda, Chiba 278–8510, Japan

**Keywords:** MAP kinase, MPK4, *Nicotiana*, *tabacum*, ozone, stomata, TEY motif.

## Abstract

The mitogen-activated protein kinases (MAPKs/MPKs) are important factors in the regulation of signal transduction in response to biotic and abiotic stresses. Previously, we characterized a MAPK from tobacco, *Nicotiana tabacum* MPK4 (NtMPK4). Here, we found a highly homologous gene, *NtMPK4-like* (*NtMPK4L*), in tobacco as well as other species in Solanaceae and Gramineae. Deduced amino acid sequences of their translation products carried MEY motifs instead of conserved TXY motifs of the MAPK family. We isolated the full length *NtMPK4L* gene and examined the physiological functions of NtMPK4L. We revealed that NtMPK4L was activated by wounding, like NtMPK4. However, a constitutively active salicylic acid-induced protein kinase kinase (SIPKK^EE^), which phosphorylates NtMPK4, did not phosphorylate NtMPK4L. Moreover, a tyrosine residue in the MEY motif was not involved in NtMPK4L activation. We also found that NtMPK4L-silenced plants showed rapid transpiration caused by remarkably open stomata. In addition, NtMPK4L-silenced plants completely lost the ability to close stomata upon ozone treatment and were highly sensitive to ozone, suggesting that this atypical MAPK plays a role in ozone tolerance through stomatal regulation.

## Highlight

Tobacco MPK4L is activated by wounding and ozone exposure in a pathway independent to that of MPK4 and plays a role in the control of stomatal aperture.

## Introduction

Mitogen-activated protein kinases (MAPKs/MPKs) are conserved serine/threonine protein kinases present in all eukaryotes (Avruch, 2007). There are several types of MAPKs, with 20 genes encoding putative MAPKs found in the Arabidopsis genome (MAPK group, 2002). In plants, MAPKs are known to be involved in physiological functions and in responses to numerous biotic and abiotic stresses. For example, in Arabidopsis, AtMPK3 and AtMPK6 regulate various biological functions such as funicular guidance of pollen tubes, immunity, salt tolerance, and responses to hyperosmolarity and low temperature (Ichimura *et al.*, 2000; Guan *et al.*, 2014; Pecher *et al.*, 2014; Pérez-Salamó *et al.*, 2014). In tobacco, wound-induced protein kinase (WIPK) and salicylic acid-induced protein kinase (SIPK), which are orthologs of AtMPK3 and AtMPK6, respectively, are involved in responses to wounding and ozone (Seo *et al.*, 1995; Zhang and Klessig, 1998; Samuel and Ellis 2002; Seo *et al.*, 2007; Katou *et al.*, 2013). Another MAPK, MPK4, is one of the most characterized MAPKs in plants. AtMPK4 is a regulator of biotic stress responses dependent on salicylic acid (SA) and jasmonate (JA) (Petersen *et al.*, 2000). By analysis of *mpk4* mutants, it was also found that MPK4 is involved in osmotic stress-response pathways, male-specific meiotic cytokinesis and cortical microtubule organization in Arabidopsis (Droillard *et al.*, 2004; Beck *et al.*, 2010; Zeng *et al.*, 2011). Moreover, AtMPK4 is also a regulator of photosynthesis, reactive oxygen species metabolism and growth (Gawroński *et al.*, 2014).

It is known that MAPKs are enzymatically activated by the phosphorylation of threonine and tyrosine residues in the TXY motif by MAPK kinase (MAPKK/MEK/MKK) (Anderson *et al.*, 1990). In tobacco, NtMEK2 acts as an upstream kinase of WIPK and SIPK (Yang *et al.*, 2001). In Arabidopsis, both AtMPK3 and AtMPK6 are activated by a constitutively active mutant of MKK4, MKK5, and MKK9 *in vitro* (Asai *et al.*, 2002; Xu *et al.*, 2008). Moreover, other MAPKs in Arabidopsis, AtMPK6, AtMPK10, and AtMPK20, are reported to be phosphorylation substrates of a constitutive active mutant of MKK9 (Lee *et al.*, 2008). NtMPK4 is also phosphorylated by a constitutive active mutant of SIPK kinase (SIPKK^EE^) (Gomi *et al.*, 2005; Wu *et al.*, 2014). Lee *et al.* (2008) also indicated that constitutively active forms of MKK1 and MKK2 can phosphorylate MPK4 in Arabidopsis.

It has been reported that *OsMPK2* is homologous to *AtMPK4*, but its deduced amino acid sequence carries an MEY motif instead of a TXY motif (Rohila and Yang, 2007). In addition, Stulemeijer *et al.* (2007) indicated that tomato had two genes, *SlMPK5* and *SlMPK6*, homologous to *AtMPK4*, and their translation products had TXY and MEY motifs, respectively. We have already reported that NtMPK4 carries a TXY motif (Gomi *et al.*, 2005), and this protein is thought to be an ortholog of SlMPK5 in tobacco.

In this study, we found a homologous gene to *SlMPK6* in tobacco, which we named *NtMPK4-like* (*NtMPK4L*), and its translation product had an MEY motif instead of a TXY motif. No functional information on MEY-type MAPKs has been reported so far, although several MEY-type MAPK genes were reported in potato, *Brachypodium*, millet, *Sorghum*, and maize in addition to tomato and rice (Mohanta *et al*, 2015). Therefore, to elucidate the physiological functions of NtMPK4L, we isolated the full-length *NtMPK4L* cDNA from tobacco leaves. We found that NtMPK4L was activated upon wounding and ozone exposure, although it lacks the TXY motif. Moreover, analysis of NtMPK4L-silenced plants revealed that NtMPK4L are involved in stomatal regulation and ozone tolerance.

## Materials and methods

### Plant materials and treatment

Plants of tobacco (*Nicotiana tabacum* cv. Samsun NN) and tomato (*Solanum lycopersicum* L. cv. MicroTom) were grown in a growth room controlled at 25 °C in 16h light/8h dark conditions. NtMPK4-silenced transgenic plants produced with inverted repeat (IR) constructs using RNA silencing technology, named NtMPK4-IR plants, were produced previously (Gomi *et al.*, 2005). For wounding treatment, leaf discs were floated on tap water for the indicated time periods.

### Cloning of NtMPK4L-1, construction of the NtMPK4L-silencing vector and NtMPK4L-silenced transgenic plants

Leaves at 1, 10, or 30min after wounding were combined and total RNA was extracted using TRIzol (Life Technologies, Tokyo, Japan) in accordance with the manufacturer’s instructions. An *NtMPK4L-1* cDNA fragment was amplified from total RNA using degenerate primers NtMMPK4L Dege 5′ and NtMMPK4L Dege 3′. The PCR product was further amplified using primers NtMPK4L 5′ and NtMPK4L 3′, and then the resulting cDNA fragment was inserted into pGEM-T easy vector (Promega, Madison, WI, USA).

For construction of the *NtMPK4L*-silencing vector, a 5′ fragment (68bp of the 5′-UTR and 63bp of the ORF) or 3′ fragment (33bp with the stop codon and 126bp of 3′-UTR) of the *NtMPK4L* cDNA was amplified using primers; 5′+3′UTR BamHI 5′ and 5′UTR+3′UTR TR Rv or 3′UTR Fw and 5′+3′UTR XhoI 3′, respectively (see Supplementary Fig. S1 at *JXB* online). Both fragments were connected by recombinant PCR using primers 5′+3′UTR BamHI 5′ and 5′+3′UTR XhoI 3′. The resulting DNA fragment was inserted into the *Bam*HI and *Xho*I sites of pBluescript SK+. A DNA fragment carrying *Sac*I and *Kpn*I sites at the 5′ and 3′ termini was further amplified from the plasmid using primers 5′+3′UTR SacI 5′ and 5′+3′UTR KpnI 3′. Each fragment was inserted into pBE2113 carrying a GUS fragment (from 821 to 1809) (Mitsuhara *et al.*, 1996) at the *Bam*HI and *Xho*I sites or *Sac*I and *Kpn*I sites, respectively. The resulting plasmid was introduced into tobacco plants using *Agrobacterium tumefaciens* LBA4404 as described by Gomi *et al.* (2005). Second generation plants were used as NtMPK4L-silenced plants (NtMPK4L-IR plants) for this study.

Detailed information on all primers is listed in Supplementary Table S1.

### Quantitative real-time RT-PCR (qPCR)

Total RNA was extracted from leaf discs using TRIzol (Life Technologies) in accordance with the manufacturer’s instructions. qPCR was performed using iScript^TM^ cDNA synthesis kit (Bio-Rad Laboratories, Tokyo, Japan) and iQ^TM^ SYBR Green Supermix (Bio-Rad Laboratories) in accordance with the manufacturer’s instructions. Actin (AB158612) was used for normalization. Primer information for those used in this study is listed in Supplementary Table S1.

### Making antibodies against NtMPK4L, SlMPK5, and SlMPK6

An N-terminal peptide of NtMPK4L (SSSGDHSSNIRG) or C-terminal peptide of SlMPK5 (TVNFNPDSTH) or SlMPK6 (WREAAKFNPDPTH) was used to make a peptide antibody against NtMPK4L (see Supplementary Figs S2, S3). As a quality check of the anti-NtMPK4L antibody, each ORF of *NtMPK4* or *NtMPK4L-1* was amplified using primer sets; NtMPK4 NdeI 5′ and NtMPK4 BamHI 3′ or NtMPK4L-1 NdeI 5′ and NtMPK4L-1 BamHI 3′, respectively. For a quality check of the antibodies against SlMPK5 or SlMPK6, the ORF of *SlMPK5* or *SlMPK6* was amplified using primer sets; 5′ NdeI-SlMPK5 and 3′ BamHI-SlMPK5 or 5′ NdeI-SlMPK6 and 3′ BamHI-SlMPK6, respectively. The resulting *SlMPK6* fragment was cloned into a pCR-TOPO vector from a TA cloning kit (Thermo Fisher Science) producing TOPO-SlMPK6. After each fragment of *NtMPK4*, *NtMPK4L*, *SlMPK5,* or *SlMPK6* carrying *Nde*I and *Bam*HI sites was produced by digestion with *Nde*I and *Bam*HI, the fragment was inserted into the *Nde*I and *Bam*HI sites of a pET15b vector carrying a histidine (His) tag. *His-NtMPK4*, *His-NtMPK4L-1*, *His-SlMPK5,* and *His-SlMPK6* plasmids were introduced into *Escherichia coli*. His-NtMPK4, His-NtMPK4L-1, His-SlMPK5, or His-SlMPK6 was purified using Ni sepharose^TM^ high performance (GE Healthcare, Tokyo, Japan) in accordance with the manufacturer’s instructions. The purified proteins were used to check the cross-reactivity of anti-NtMPK4 and anti-NtMPK4L (Supplementary Fig. S4) or anti-SlMPK5 and anti-SlMPK6 antibodies (Supplementary Fig. S5). Primer information for those used in this study is listed in Supplementary Table S1.

### Immune complex kinase assay and in vitro kinase assay

An immune complex kinase assay was performed using anti-NtMPK4, anti-NtMPK4L, anti-SlMPK5, or anti-SlMPK6 antibodies as described previously (Seo *et al.*, 1999). An anti-NtMPK4 antibody was produced previously (Gomi *et al*, 2005).

To prepare recombinant GST-SlMKK1, an ORF of *MKK1* was amplified using primer set; 5′ EcoRI-SlMKK1 and 3′ SalI-SlMKK1. The resulting fragment was cloned into a pCR-TOPO vector of a TA cloning kit (Thermo Fisher Science) producing TOPO-SlMKK1. To prepare GST-SlMKK1^EE^, site-directed mutagenesis was performed from TOPO-SlMKK1 using primer sets; SlMKK1 ^EE^ Fw and SlMKK1 ^EE^ Rv. SlMKK1 or SlMKK1 ^EE^ fragment was digested with *Eco*RI and *Sal*I, and then inserted into pGEX4T-1 vector carrying a glutathione sepharose (GST) tag (GE Healthcare, Tokyo, Japan). The resulting plasmids were introduced into *E. coli*. GST-SlMKK1 or GST-SlMKK1^EE^ was purified using Glutathione Sepharose 4B (GE Healthcare) in accordance with the manufacturer’s instructions.

The *in vitro* kinase assay was performed as described by Gomi *et al.* (2005). His-NtMPK4, His-NtMPK4L-1, His-SlMPK5 or His-SlMPK6 (500ng), and MBP (5 μg, Sigma-Aldrich Inc, Osaka, Japan) were incubated with GST-SIPKK, GST-SIPKK^EE^, GST-SlMKK1, or GST-SlMKK1^EE^ (50ng). Recombinant GST-SIPKK or GST-SIPKK^EE^ was as described previously (Gomi *et al.*, 2005). To prepare an expression vector of SlMPK6^Y203F^, TOPO-SlMPK6 was used for site-directed mutagenesis using primer sets; SlMPK6YF Fw and SlMPK6YF Rv. Each ORF of *SlMPK6* or *SlMPK6* ^*Y203F*^ was amplified using SlMPK6-BamHI Fw and SlMPK6-XhoI Rv from TOPO-SlMPK6 or TOPO-SlMPK6^Y203F^ as a template, respectively. Each of *SlMPK6* or *SlMPK6* ^*Y203F*^ was inserted into the *Bam*HI and *Xho*I sites of a pEl2Ω::HA vector (Kobayashi *et al.*, 2011) producing HA-SlMPK6 or HA-SlMPK6^Y203F^. To prepare an expression vector of HA-sGFP, sGFP was amplified from pGWB5 (Nakagawa *et al.*, 2007) using EcoRI-sGFP and sGFP-XhoI primers. The PCR product was digested with *Eco*RI and *Xho*I and then inserted into pENTR^TM^3C vector (Thermo Fisher Scientific, Kanagawa, Japan). The produced plasmid was transferred to a destination vector pGWB15 carrying a 3xHA tag (Nakagawa *et al.*, 2007). HA-SlMPK6, HA-SlMPK6^Y203F^, or HA-sGFP was transformed into *A. tumefaciens* strain GV3101 and agroinfiltrated into tobacco leaves.

Primer information for those used in these studies is listed in Supplementary Table S1.

### Water loss, ozone exposure, and stomatal aperture

The measurement of water loss was performed as described by Gomi *et al.* (2005). Leaves detached from 7-week-old plants were used.

Ozone exposure was basically performed as described by Gomi *et al.* (2005). For the observation of ozone tolerance, leaves detached from 6-week-old plants in plastic pots treated with tap water were exposed to 0.2 μl l^−1^ ozone for 6h and then transferred to a growth room without ozone for 20h.

For stomatal aperture or immune complex kinase assay with exposure to ozone, leaves were treated with ozone for 4h or 1h as described above. For the examination of stomatal aperture, the width and length of at least 70 stomatal pores were measured as described by Gomi *et al.* (2005). The epidermis was observed using a stereoscopic microscope SMZ-U (Nikon Corp., Tokyo, Japan) or SMZ1000 (Nikon Corp., Tokyo, Japan) and photo images were captured using a Digital Sight DS-L1 or Cool Pix P310 (Nicon Corp.) camera for normal or ozone exposed plants, respectively.

### Subcellular localization

For construction of the synthetic green fluorescent protein (sGFP)-fused vector, a sGFP fragment carrying *Spe*I and *Sac*I sites was amplified using primers SpeI-sGFP-F and SacI-sGFP-R. The resulting fragment was inserted into a pEl2Ω-MCS vector (Ohtsubo *et al.*, 1999) to produce pEl2Ω-sGFP. An NtMPK4 or NtMPK4L-1 fragment was amplified using primer sets XbaI-NtMPK4-F and EcoRI-NtMPK4-R or XbaI-NtMPK4L-1-F and EcoRI-NtMPK4L-1-R, respectively. The DNA fragments were inserted into the pEl2Ω-sGFP vector, and the resulting plasmids were introduced into *A. tumefaciens* GV3101 and transiently expressed by agroinfiltration. GFP images and intrinsic fluorescence were captured using a confocal laser scanning microscope FV-300 (Olympus Corp.) with Fluoview software (Olympus Corp.). Primer information for those used in this study is listed in Supplementary Table S1.

## Results

### Tobacco has a MPK4-like (NtMPK4L) gene that carries an MEY motif

**Fig. 1.**
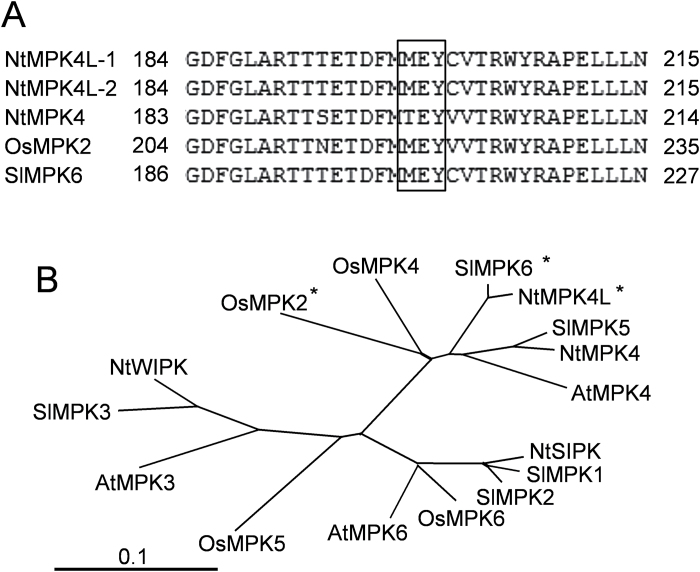
Comparison of MAPK/MPK and MAPK-related proteins. (A) Comparison of the TXY motif and corresponding sequences (square) among NtMPK4L-1, NtMPK4L-2, NtMPK4, OsMPK2, and SlMPK6. (B) Phylogenetic relationship of selected MAPK and MAPK-related proteins from tobacco, tomato, rice, and Arabidopsis. ClustalW (http://clustalw.ddbj.nig.ac.jp/top-j.htmlam) was used to construct the tree by phylogenetic analysis. Asterisks indicate proteins carrying the MEY motif instead of the TXY motif. The sequence information, except that for NtMPK4L of from of tobacco and Arabidopsis, was obtained from Ahlfors *et al.* (2004). The sequence information of tomato and rice was obtained from the PSGB tomato genome database (http://pgsb.helmholtz-muenchen.de/plant/tomato/) and Knowledge-based Oryza Molecular Biological Encyclopedia (http://cdna01.dna.affrc.go.jp/cDNA/), respectively.

It has already been reported that Solanaceae such as tomato SlMPK6 and potato StMPK4-1 and Gramineae such as rice OsMPK2, maize ZmMPK4-2, millet SiMPK4-2, *Brachypodium* BdMPK4-2, and *Sorghum* SbMPK4-2 have MEY-type MAPKs, unlike in Brassicaceae and Leguminosae (Rohila and Yang, 2007; Stulemeijer *et al*, 2007; Mohanta *et al*, 2015). Our previous report showed that NtMPK4 is an ortholog of SlMPK5 carrying a TXY motif in tobacco (Gomi *et al*, 2005). Because tomato belongs to the Solanaceae and also has a MAPK-like protein SlMPK6 carrying a MEY motif as described above, tobacco may have a gene homologous to *SlMPK6*. To clarify this, we tried to clone the *NtMPK4-like* (*NtMPK4L*) gene from tobacco. As expected, two *NtMPK4L* genes *NtMPK4L-1* (LC061268) and *NtMPKL-2* (LC063771), which seemed to originate from ancestral *Nicotiana sylvestris* and *N. tomentosiformis* were found, and both had conserved MEY motifs instead of TXY motifs (Fig. 1A, Supplementary Figs S1, S2). Because only two amino acids were different between NtMPK4L-1 and NtMPK4L-2 (see Supplementary Fig. S2), NtMPK4L-1 was used for further experiments. Next, we compared the amino acid sequences of NtMPK4L and other well known MAPKs using a phylogenetic tree (Fig. 1B). NtMPK4L was classified into the same clade as SlMPK6, suggesting that it is probably an ortholog of SlMPK6.

Because NtMPK4L was divided into a distinct clade from NtMPK4, the subcellular localization may be different. Thus, the localization of GFP-fused NtMPK4L-1 and NtMPK4 was analyzed using confocal laser microscopy (Supplementary Fig. S6). As a result, both proteins showed quite similar localization in both the nucleus and cytosol.

### NtMPK4L activity is increased by wounding

**Fig. 2.**
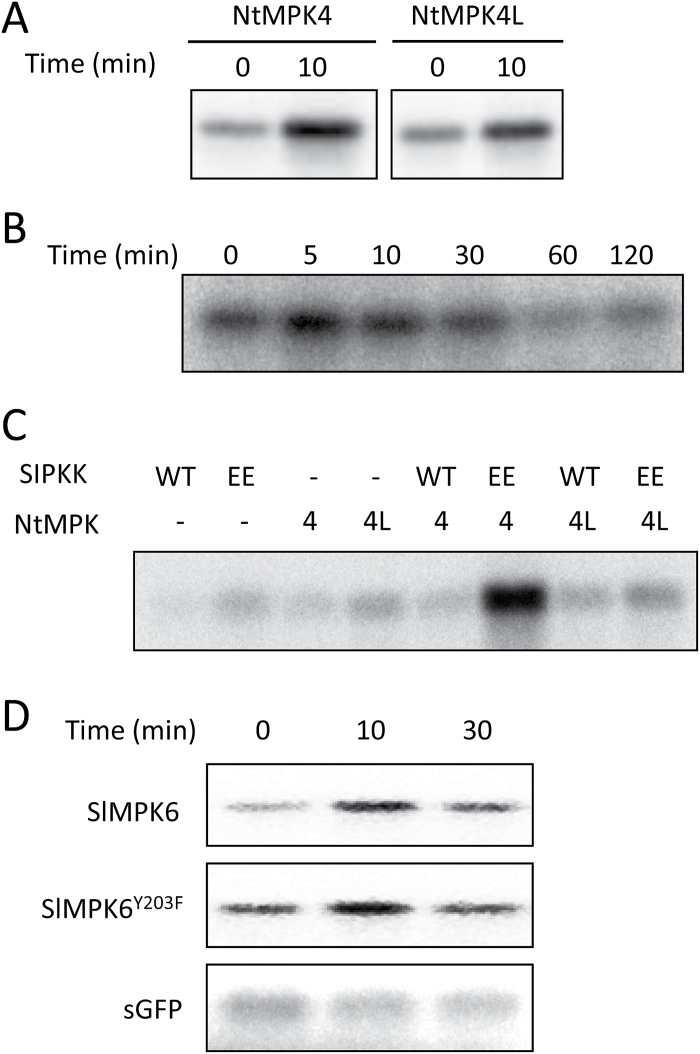
NtMPK4L activity is upregulated by wounding. Phosphorylation of MBP as a substrate was detected by autoradiography. (A) Tobacco leaves were wounded using a cork borer. After 0 and 10min, the leaf discs were used for the measurement of NtMPK4 or NtMPK4L activity. (B) Tobacco leaves were wounded using a cork borer. After 0, 5, 10, 30, 60 and 120min, the leaf discs were used for the measurement of NtMPK4L activity. (C) *In vitro* activation of His-NtMPK4 or His-NtMPK4L by GST-SIPKK (WT) or GST-SIPKK^EE^ (EE) was examined. (D) Tobacco leaves expressing HA-SlMPK6 or HA-SlMPK6^Y203F^ by agroinfiltration were wounded using a cork borer. After 0, 10 and 30min, immune complexes prepared from the leaf discs were used for the measurement of SlMPK6 or SlMPK6^Y203F^ activity. Tobacco leaves expressing HA-sGFP (sGFP) by agroinfiltration were used as negative control.

The kinase activity of NtMPK4L was subsequently examined. Antiserum against an N-terminal peptide sequence of NtMPK4L (see Supplementary Fig. S2) was raised, and its specificity was confirmed using a recombinant histidine-fused NtMPK4L (His-NtMPK4L) (Supplementary Fig. S4). As expected, the anti-NtMPK4L antibody detected only His-NtMPK4L protein and not His-NtMPK4. An immune complex kinase assay using the anti-NtMPK4L antibody and myelin basic protein (MBP) as an artificial substrate indicated that NtMPK4L was activated within 10min of wounding, similar to NtMPK4 (Fig. 2A, Supplementary Fig. S7A), for which the results were similar in SlMPK5 and SlMPK6 (Supplementary Fig. S8A). By a time-course experiment of the immune complex kinase assay (Fig. 2B, Supplementary Fig. S7B), it was shown that the kinase activity of NtMPK4L transiently increased at 5min and then decreased in wounded tobacco leaves, a pattern similar to the kinase activity of NtMPK4 (Gomi *et al*, 2005). There was no significant difference of the expression level in *NtMPK4L* transcript (Supplementary Fig. S7C). It is known that phosphorylation is necessary for the activation of NtMPK4. Because NtMPK4 is a target protein of SIPK kinase (SIPKK), NtMPK4L could be phosphorylated by it as well, although it has an MEY motif instead of a TXY motif. To verify this hypothesis, recombinant MPK4L-1 or MPK4 proteins were treated with wild-type SIPKK or constitutively active SIPKK^EE^, and MBP kinase activity was assayed. As shown in Fig. 2C, NtMPK4L was activated by neither SIPKK nor SIPKK^EE^, but NtMPK4 was activated by SIPKK^EE^. To examine whether the tyrosine residue in the MEY motif is necessary for activation, we performed the immune complex kinase assay using tobacco leaves expressing tagged NtMPK4L or NtMPK4L^YF^. Unfortunately, activity was observed in neither NtMPK4L nor NtMPK4L^YF^ (data not shown). A probable reason for this result came from the low expression level of those proteins, although it is still unknown why their expression levels were poor. Therefore, we next tried to use a heterogenic expression system. To do this, HA-SlMPK6 or HA-SlMPK6^Y203F^ carrying a phenylalanine residue instead of a tyrosine residue in the MEY motif was expressed in tobacco leaves by agroinfiltration. As a result, the immune complex kinase assay indicated that there was no significant difference between SlMPK6 and SlMPK6 ^Y203F^ in their activation (Fig. 2D, Supplementary S7D). No significant alternation from 0 to 30min was observed in a netagive control sGFP, although artificial signals appeared.

### Expression level of NtMPK4L transcript and the activity of its protein were significantly repressed in NtMPK4-silenced plants

Previously, we characterized NtMPK4 using NtMPK4-silenced plants produced by introduction of an IR construct using approximately 800bp of the 5′ region of the *NtMPK4* cDNA, including sequences highly similar to *NtMPK4L* (Gomi *et al.*, 2005). However, comparison of the nucleic acid sequences of the ORFs between *NtMPK4* and *NtMPK4L* showed both sequences had high similarity (see Supplementary Fig. S1), implying that not only NtMPK4 but also NtMPK4L could be silenced in NtMPK4-silenced plants. If this is the case, NtMPK4-silenced plants should show multiple features from the silencing of both *NtMPK4* and *NtMPK4L*. Thus, to characterize NtMPK4L, NtMPK4L-silenced plants were produced using an IR construct with *NtMPK4L*-specific sequences, as described in the ‘Materials and methods’ section. The first generation of 17 independent NtMPK4L-silenced lines was selected, and the expression levels of *NtMPK4L* were determined. Five independent plants (NtMPK4L-IRs 2, 3, 5, 6 and 8) showed approximately one-tenth the expression level of the *NtMPK4L* transcript compared with a vector control plant, whereas there was no significant difference in *NtMPK4* transcript levels (Supplementary Fig. S9). The second generation of two lines, NtMPK4L-IR6 and NtMPK4L-IR8, were used as NtMPK4L-silenced plants for further experiments.

**Fig. 3.**
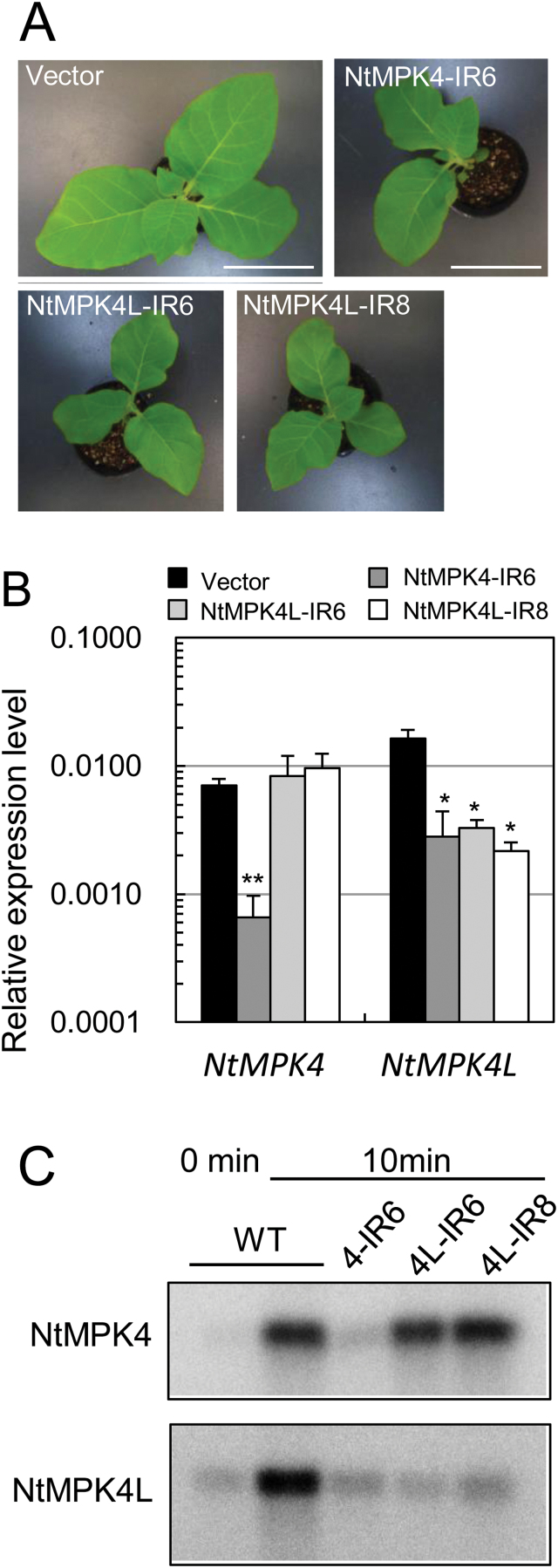
Characterization of NtMPK4-silenced and NtMPK4L-silenced plants. (A) Pictures of 1-month-old transgenic plants after sowing. Bar, 9cm. (B) Relative expression levels of *NtMPK4* and *NtMPK4L* transcripts were measured. Error bars indicate standard deviations determined from three independent biological replicates. Asterisks indicate significant differences analyzed using Student’s *t*-test compared with the relevant vector control at *P*<0.05 (*) or *P*<0.01 (**). (C) Immune complex kinase assay of NtMPK4 and NtMPK4L in wild-type (WT), NtMPK4-IR6 (4-IR6), NtMPK4L-IR6 (4L-IR6), and NtMPK4L-IR8 (4L-IR8) plants. Crude extracts were prepared from leaf discs after wounding for 0 or 10min. Phosphorylation of MBP as a substrate was detected by autoradiography. Three independent experiments were performed in all figures.

Firstly, we observed the morphological phenotype of NtMPK4L-silenced plants. As shown in Fig. 3A, both NtMPK4L-IR6 and NtMPK4L-IR8 plants showed a semi-dwarf phenotype similar to an NtMPK4-silenced plant (NtMPK4-IR6). Next, the expression level of both *NtMPK4* and *NtMPK4L* transcripts were determined by qPCR (Fig. 3B). Both NtMPK4L-IR lines showed low levels of *NtMPK4L* transcripts (approximately one-fifth or one-eighth of that in NtMPK4L-IR6 and NtMPK4L-IR8, respectively, compared with a vector control plant), but no effect on *NtMPK4*. As suspected, the expression level of *NtMPK4L* transcripts was approximately one-sixth of NtMPK4-silenced plants produced by Gomi *et al.* (2005) compared with vector control plants, in addition to the low level of *NtMPK4* transcripts, suggesting that the phenotypes exhibited in NtMPK4-silenced plants were caused by NtMPK4 and/or NtMPK4L knockdown. We tried to produce novel NtMPK4-silenced plants with normal expression levels of *NtMPK4L* transcripts using a non-coding region for IR constructs; however, no useable plant lines were obtained (data not shown).

Then, we examined wounding-induced activation of NtMPK4L and NtMPK4 in both NtMPK4-IR and NtMPK4L-IR plants (Fig. 3C, Supplementary Fig. S10). As expected, NtMPK4L was hardly activated by wounding in NtMPK4L-IR6 or NtMPK4L-IR8 plants, whereas NtMPK4 was activated in these plants at similar levels to wild-type plants. On the other hand, activation of neither NtMPK4 nor NtMPK4L was detected by wounding in NtMPK4-IR6 plants, implying that NtMPK4-silenced plant had low amounts of NtMPK4L in addition to NtMPK4 compared with wild-type plants.

### NtMPK4L is involved in stomatal regulation

**Fig. 4.**
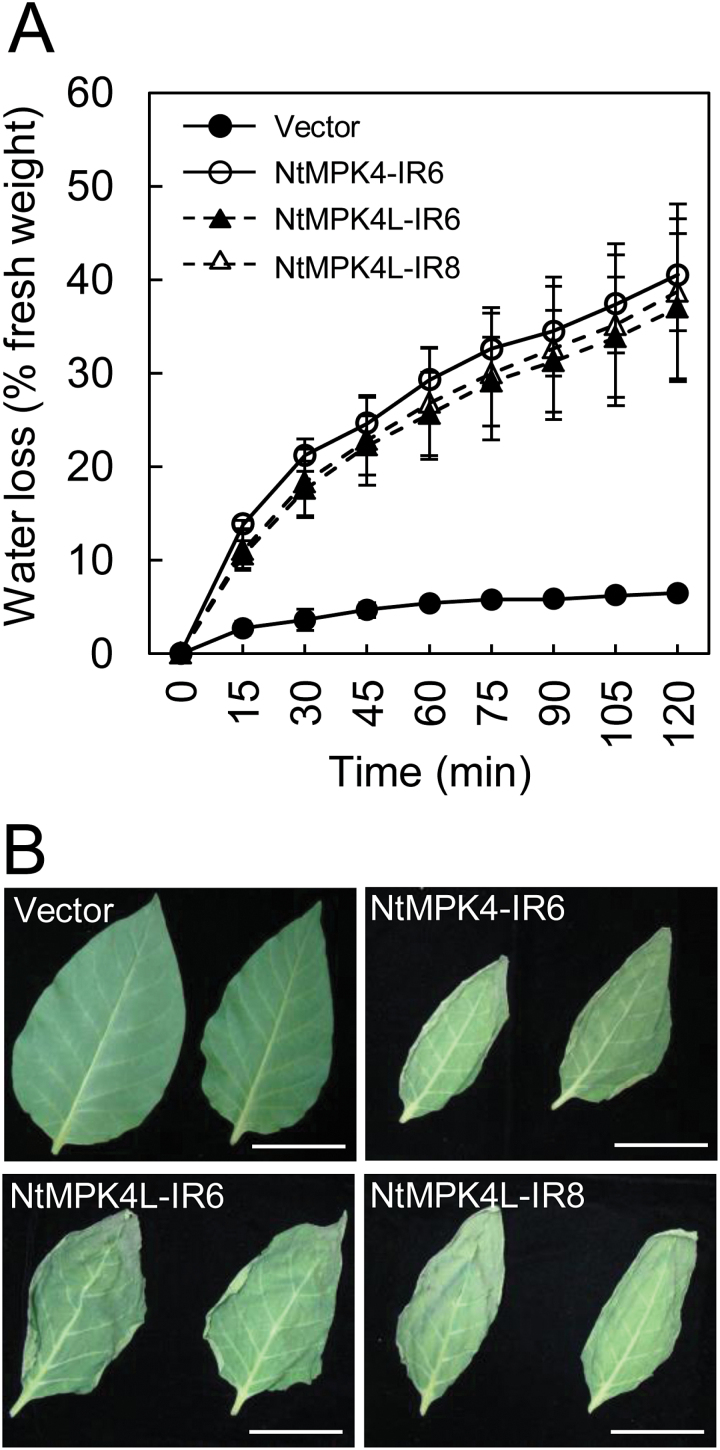
NtMPK4L-silenced plants are sensitive to water loss. (A) Tobacco leaves were left at room temperature for 120min. Water loss was calculated from fresh weight of the leaves. Error bars indicate standard deviations determined from three independent biological replicates. Three independent experiments were performed. (B) Leaf pictures were taken after leaving for 120min. Bar, 5cm.

We reported previously that NtMPK4-silenced plants showed a higher rate of water loss by transpiration from the leaves than vector control plants (Gomi *et al.*, 2005). Because NtMPK4-silenced plants also had a low expression level of *NtMPK4L* transcripts, this may be caused by the repression of *NtMPK4L* in NtMPK4-silenced plants. To consider this possibility, the kinetics of water loss by transpiration was examined using leaves of NtMPK4L-silenced plants (Fig. 4). In Fig. 4A, water loss was significantly more rapid in leaves of both NtMPK4L-IR6 and NtMPK4L-IR8 plants compared with vector control plants, as well as NtMPK4-IR6 plants. After 120min, leaves of NtMPK4L-IR6 and NtMPK4L-IR8 plants had lost 37% and 39% of their weight and had a withered shape due to accelerated water loss (Fig. 4B). This was similar to NtMPK4-IR6 plants (41%), whereas vector control plants showed only 6.5% weight loss.

**Fig. 5.**
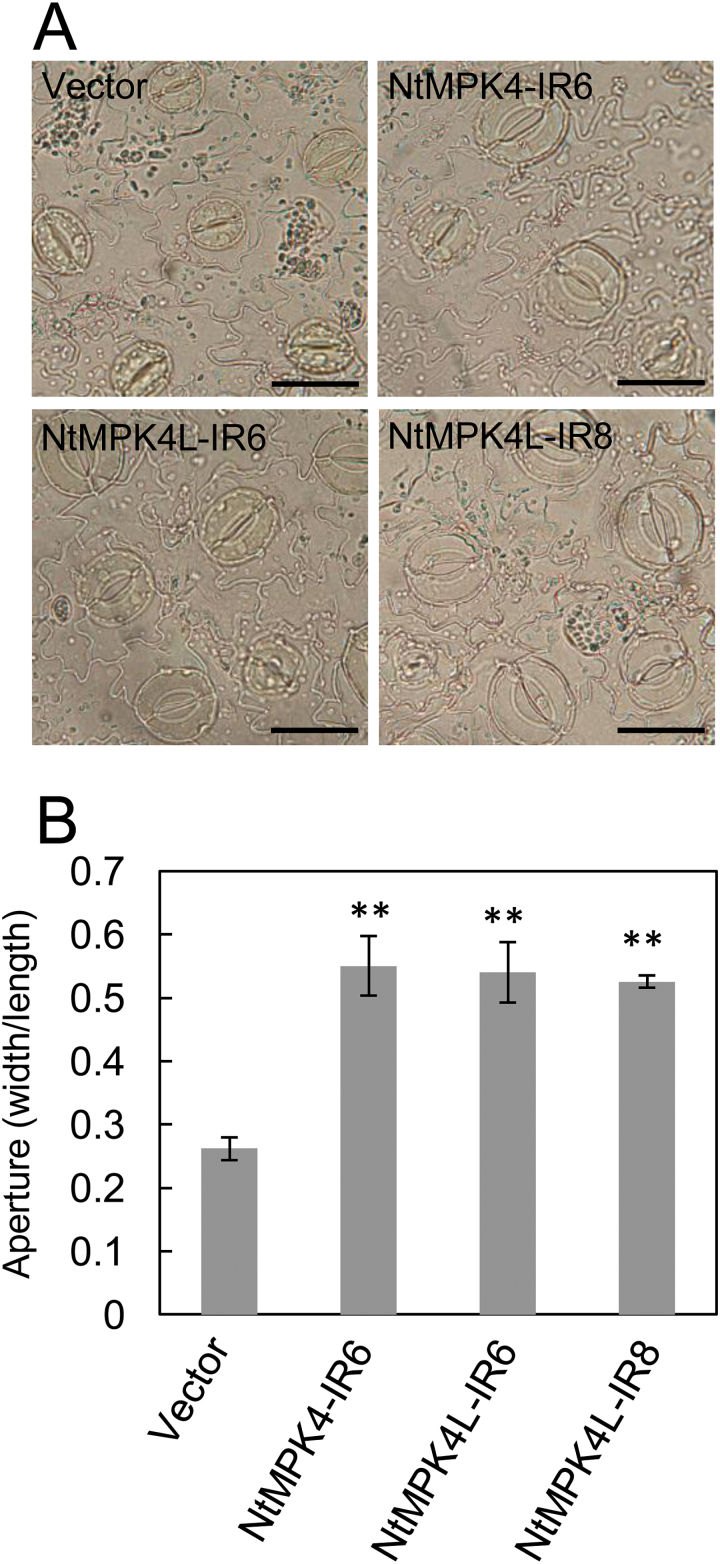
NtMPK4L-silenced plants have significantly opened stomata. (A) Epidermis was observed in NtMPK4-IR6, NtMPK4-IR, NtMPK4L-IR, and vector control plants. Bar, 5 μm. (B) Stomatal apertures (width/length) were calculated. Error bars indicate standard deviations determined from three independent biological replicates. Asterisks indicate significant differences analyzed using Student’s *t*-test compared with the relevant vector control at *P*<0.01 (**). Three independent experiments were performed.

Transpiration is regulated by opening and closing of stomata. Previously, we suggested that these high levels of water loss were caused by remarkable transpiration resulting from reduced stomatal closure in NtMPK4-silenced plants. Because the leaves from both NtMPK4L-IR lines showed significantly higher transpiration similar to NtMPK4-IR6 plants, the stomata of NtMPK4L-silenced plants may by significantly more open. As expected, both NtMPK4L-IR6 and NtMPK4L-IR8 plants had significantly larger stomatal apertures (width/length) than vector control plants and similar to NtMPK4-IR6 plants (Fig. 5).

### NtMPK4L is involved in ozone tolerance

**Fig. 6.**
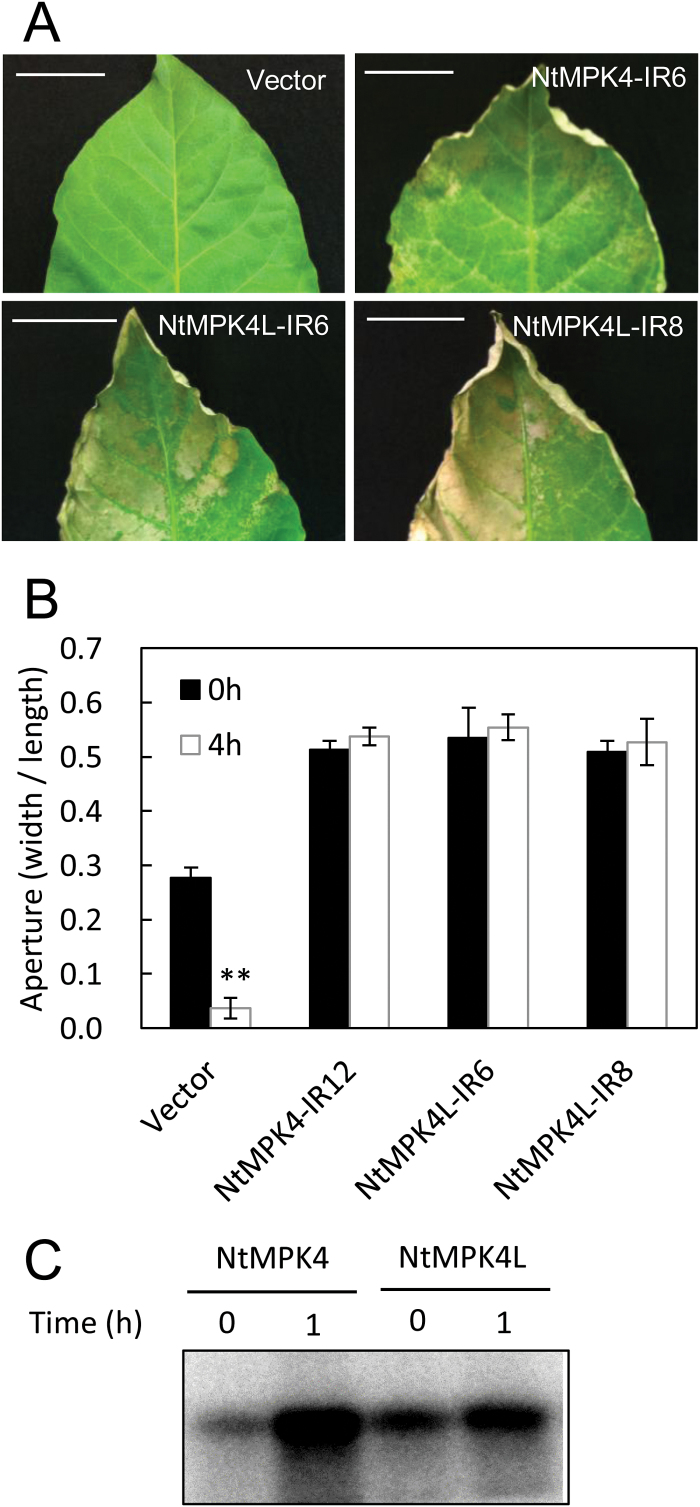
NtMPK4L is involved in ozone tolerance. (A) Visible injury on the leaves from NtMPK4-IR6, NtMPK4L-IR6, NtMPK4L-IR8, and vector control plants were observed after one day of ozone exposure. Bar, 4cm. (B) Stomatal apertures (width/length) of the leaves from NtMPK4-IR12, NtMPK4L-IR6, NtMPK4L-IR8, and vector control plants were calculated before (0h) or after (4h) ozone exposure. Error bars indicate standard deviations determined from three independent biological replicates. Asterisks indicate significant differences analyzed using Student’s *t*-test compared between before and after ozone exposure of each plant line at *P*<0.01(**). (C) Wild-type tobacco leaves were exposed by ozone. After 0 and 1h, immune complexes prepared from the leaf discs were used for the measurement of NtMPK4 or NtMPK4L activity. Three independent experiments were performed in all figures.

Our previous work showed that NtMPK4-silenced plants had high sensitivity to ozone exposure because of their wider stomatal apertures (Gomi *et al.*, 2005). Thus, NtMPK4L-silenced plants may be damaged by ozone exposure. To verify this hypothesis, detached leaves were exposed to ozone. As shown in Fig. 6A, both NtMPK4L-IR6 and NtMPK4L-IR8 plants showed severe damages, including cell death, after ozone exposure similar to NtMPK4-IR6 plants, but vector control plants were hardly affected. It is known that ozone enters through open stomatal apertures and plants close their stomata in response to ozone exposure to prevent ozone damage (Vsinonrn & Kangasjärvi, 2015). Because NtMPK4L-silenced plants were highly sensitive to ozone exposure compared with vector control plants, the stomata may not close in response to ozone exposure. To examine this hypothesis, the stomatal apertures of detached leaves were compared before and after ozone exposure (Fig. 6B). As expected, both NtMPK4L-IR6 and NtMPK4L-IR8 plants maintained remarkably open stomata after ozone exposure, at similar levels to that before ozone exposure, as did NtMPK4-IR12 plants, with reduced expression levels of both *NtMPK4* and *NtMPK4L* transcripts (see Supplementary Fig. S11). On the other hand, vector control plants significantly closed their stomata in response to ozone exposure. Next, we examined whether NtMPK4L activity was altered by ozone exposure. As shown in Fig. 6C, the activity of not only NtMPK4 but also NtMPK4L increased in response to ozone exposure, although any significant alternation was obtained in neither the expression of their transcripts nor total protein amount by ozone exposure (Supplementary Fig. S12).

## Discussion

In this study, we found that MPK4L proteins carrying an MEY motif are present in tobacco and other species in Solanaceae as well as Gramineae. Although NtMPK4L does not have a typical TXY motif, nor is it activated by SIPKK^EE^, a constitutively active MAPKK for NtMPK4, it was activated by wounding. As well as tobacco, a tomato NtMPK4L ortholog, SlMPK6, was not phosphorylated by a constitutively active form of SIPKK ortholog, SlMKK1, unlike SlMPK5 (see Supplementary Fig. S8). Because the MPK family needs its phosphorylation to be activated in general, MPK4L proteins might be phosphorylated by a distinct cascade(s) with another type of MAPKK or other kinase, although this remains to be elucidated in the future. Notably, no other type of MAPK-like protein apart from MPK4L has been found in plants so far, also implying that MPK4L has a non-canonical cascade and/or function.

NtMPK4L-silenced plants showed abnormal opening of stomata with rapid transpiration at a similar level to NtMPK4-silenced plants. NtMPK4-silenced plants have reduced expression levels of both *NtMPK4* and *NtMPK4L*. Thus, NtMPK4L is likely more important for stomatal closure than NtMPK4, although a contribution by NtMPK4 cannot be ruled out. Ozone is known to cause cell damage in plants by entering into mesophyll tissue through open stomata and diffusing through the inner air spaces. Unlike vector control plants, NtMPK4L-silenced plants did not close their stomata in response to ozone exposure at a similar level to NtMPK4-silenced plants. Thus, the sensitivity to ozone exposure is probably related to the abnormal opening of stomata in NtMPK4L-silenced plants. Our previous study reported that NtMPK4-silenced plants were sensitive to ozone exposure (Gomi *et al.*, 2005). In this study, NtMPK4L-silenced plants showed sensitivity to ozone exposure in addition to water loss and stomatal apertures at similar levels to NtMPK4-silenced plants. Moreover, NtMPK4L was activated as well as NtMPK4 by ozone exposure. These findings implied that NtMPK4L contributes to regulate ozone tolerance.

Taken together, our results suggested that tobacco has a MPK4 paralog, MPK4L, which is highly homologous to MPK4 despite the lack of a TXY motif. MPK4L was activated upon wounding and ozone exposure as well as MPK4, but the mechanism of activation might be different from each other. It would be interesting to investigate how and why these two kinases with different activation mechanisms but similar physiological roles have emerged, and this will be a focus for future studies.

## Supplementary data

Supplementary data are available at *JXB* online.

Figure S1. Comparison of the nucleotide sequences in open-reading frames among NtMPK4, NtMPK4L-1, and NtMPK4L-2.

Figure S2. Comparison of the amino acid sequences of NtMPK4L-1, NtMPK4L-2, and NtMPK4.

Figure S3. Comparison of the amino acid sequences of SlMPK5 and SlMPK6.

Figure S4. Quality test of the anti-NtMPK4L antibody.

Figure S5. Quality test of the anti-SlMPK5 and anti-SlMPK6 antibodies.

Figure S6. Subcellular localization of sGFP-fused NtMPK4 and NtMPK4L-1 in *N. tabacum*.

Figure S7. Results of CBB staining and expression level of the *NtMPK4L* transcript on crude extracts prepared from wounded tobacco leaves.

Figure S8. SlMPK5 and SlMPK6 activity in wounded tobacco leaves, and *in vitro* activation of His-SlMPK5 or His-SlMPK6 by GST-SIMKK1 or GST-SIMKK1^EE^.

Figure S9. The relative expression levels of *NtMPK4L* and *NtMPK4* transcripts measured in first-generation NtMPK4L-silenced plants.

Figure S10. Results of CBB staining on wounded leaves from wild-type (WT), NtMPK4-IR6 (4-IR6), NtMPK4L-IR6 (4L-IR6), and NtMPK4L-IR8 (4L-IR8) plants.

Figure S11. Relative expression levels of *NtMPK4* and *NtMPK4L* transcripts for NtMPK4-IR12, NtMPK4L-IR6, and NtMPK4L-IR8 plants.

Figure S12. Separation of crude extracts stained with CBB by SDS-PAGE, and relative expression levels of *NtMPK4* and *NtMPK4L* transcripts of wild-type tobacco leaves after exposure to ozone.

Table S1. Primers used in this study.

Supplementary Data

## Abbreviations:

IR: inverted repeat
JA: jasmonic acid
MAPK/MPK: mitogen-activated protein kinase
MAPK4L: MAPK4-like
MAPKK/MEK/MKK: mitogen-activated protein kinase kinase
MBP: myelin basic protein
SIPK: salicylic acid-induced protein kinase
SIPKK: salicylic acid-induced protein kinase kinase.
