# Supplementary material for: Mitogen-activated protein kinase 4-like carrying an MEY motif instead of a TXY motif is involved in ozone tolerance and regulation of stomatal closure in tobacco
Source: J Exp Bot. 2016 Apr 28;67(11):3471–9. doi: 10.1093/jxb/erw173 (PMC4892734; doi:10.1093/jxb/erw173)
Supplement: Supplementary Data [file supp_67_11_3471__index.html]

Mitogen-activated protein kinase 4-like carrying an MEY motif instead of a TXY motif is involved in ozone tolerance and regulation of stomatal closure in tobacco — Mitogen-activated protein kinase 4-like carrying an MEY motif instead of a TXY motif is involved in ozone tolerance and regulation of stomatal closure in tobacco — Supplementary Data 

# Mitogen-activated protein kinase 4-like carrying an MEY motif instead of a TXY motif is involved in ozone tolerance and regulation of stomatal closure in tobacco

## Supplementary Data

Data files

- supplementary\_figures\_S1\_S12\_tables\_S1.pdf - Supplementary Data
