# Supplementary material for: Mitogen-activated protein kinase 4-like carrying an MEY motif instead of a TXY motif is involved in ozone tolerance and regulation of stomatal closure in tobacco
Source: J Exp Bot. 2016 Apr 28;67(11):3471–9. doi: 10.1093/jxb/erw173 (PMC4892734; doi:10.1093/jxb/erw173)
Supplement: Supplementary Data [file supp_erw173_supplementary_figures_S1_S12_tables_S1.pdf]

Mitogen-activated protein kinase 4-like carrying a MEY motif instead of a TXY motif is involved in ozone tolerance and regulation of stomatal closure

Yuki Yanagawa[1], Hiroshi Yoda[1], Kohei Osaki[1], Yuta Amano[2], Mitsuko Aono[3], Shigemi Seo[1], Kazuyuki Kuchitsu[2], Ichiro Mitsuhara[1]*

Supplementary Figure S1. Comparison of the nucleotide sequences in open-reading frames among NtMPK4, NtMPK4L-1, and NtMPK4L-2. Bars indicate the regions used as IR constructs to generate NtMPK4L-silenced plants.

Supplementary Figure S2. Comparison of the amino acid sequences of NtMPK4L-1, NtMPK4L-2, and NtMPK4. A bar indicates the region used as an antigen in the production of the anti-NtMPK4L antibody. A square indicates the MEY and TXY motifs.

Supplementary Figure S3. Comparison of the amino acid sequences of SlMPK5 and SlMPK6. Bars indicate that sequence regions used as antigens against the anti-SlMPK5 and anti-SlMPK6 antibodies. A square indicates the MEY and TXY motifs.

Supplementary Figure S4. Quality test of the anti-NtMPK4L antibody. (A) SDS-PAGE analysis was performed using purified His-NtMPK4L-1 and His-NtMPK4 proteins. (B) Immunoblot analysis was performed using the anti-NtMPK4L antibody.

Supplementary Figure S5. Quality test of the anti-SlMPK5 and anti-SlMPK6 antibodies. (A) SDS-PAGE analysis was performed using purified His-SlMPK5 and His-SlMPK6 proteins. (B) Immunoblot analysis was performed using the anti-SlMPK5 or anti-SlMPK6 antibody.

Supplementary Figure S6. Subcellular localization of sGFP-fused NtMPK4 and NtMPK4L-1 in *N. tabacum*. Bar; 50 μm.

Supplementary Figure S7. Crude extracts prepared from wounded leaves were separated by SDS-PAGE and stained with CBB. Tobacco leaves were wounded using a cork borer. After 0

and 10 min (A) or 0, 5, 10, 30, 60 and 120 min (B), the leaf discs were used for the preparation of crude extracts. (C) The expression level of *NtMPK4L* transcript was measured at 0, 5, 10, 30, 60 and 120 min after wounding. (D) Tobacco leaves expressing HA-SlMPK6, or HA-SlMPK6^{Y203F} or HA-sGFP by agroinfiltration were wounded using a cork borer. After 0, 10 and 30 min, the leaf discs were used for the preparation of crude extracts. CBB staining or an immunoblot analysis using an anti-HA antibody was performed.

Supplementary Figure S8. SlMPK6 activity was upregulated by wounding. Phosphorylation of MBP as a substrate was detected by autoradiography. (A) Tobacco leaves were wounded using a cork borer. After 0 and 10 min, the leaf discs were used for the measurement of SlMPK5 or SlMPK6 activity. (B) *In vitro* activation of His-SlMPK5 or His-SlMPK6 by GST-SIMKK1 or GST-SIMKK1^{EE} was examined.

Supplementary Figure S9. The relative expression levels of *NtMPK4L* and *NtMPK4* transcripts were measured in first-generation NtMPK4L-silenced plants. Five NtMPK4L-IR lines were compared with vector control plants.

Supplementary Figure S10. Leaves from wild-type (WT), NtMPK4-IR6 (4-IR6), NtMPK4L-IR6 (4L-IR6), and NtMPK4L-IR8 (4L-IR8) plants were wounded using a cork borer. After 0 or 10 min, the leaf discs were used for the preparation of crude extracts. CBB staining was performed.

Supplementary Figure S11. Relative expression levels of *NtMPK4* and *NtMPK4L* transcripts were measured. Error bars indicate standard deviations determined from three independent biological replicates. Asterisks indicate significant differences from analyses using Student's t-test compared with the relevant vector control at $p<0.05$(*).

Supplementary Figure S12. Wild-type tobacco leaves were exposed by ozone. (A) After 0 and 1 h, crude extracts were prepared. The crude extracts were separated by SDS-PAGE and stained

with CBB. (B) The expression levels of both *NtMPK4* and *NtMPK4L* transcripts were measured.

Supplementary Table S1. Primers used in this study.

Supplementary Figure S1

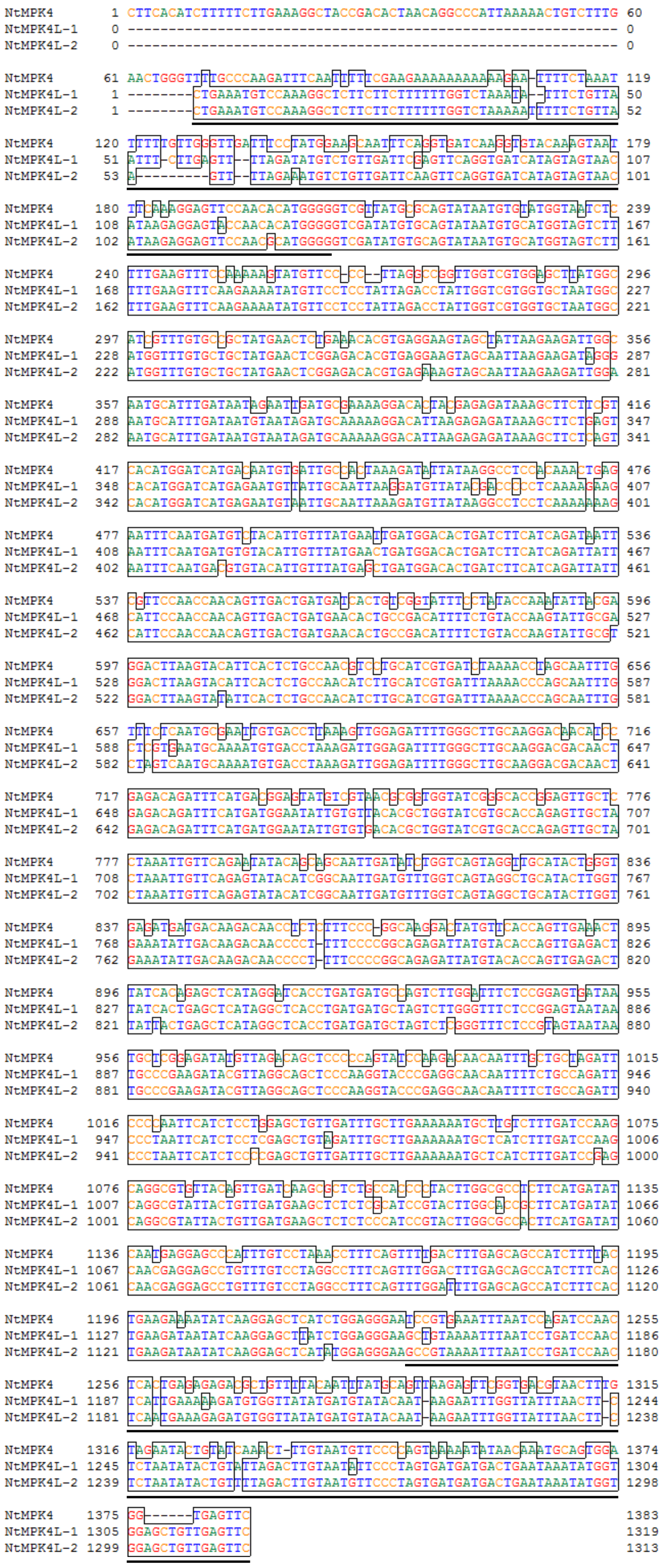

Supplementary Figure S2

```
NtMPK4L-1    1 MSVDSSS-GDHSSNIRGVPTHGGRYVQYNVHGSLFEVSRKYVPPIRPIGRGANGMVCAAM 59
NtMPK4L-2    1 MSVDSSS-GDHSSNIRGVPTHGGRYVQYNVHGSLFEVSRKYVPPIRPIGRGANGMVCAAM 59
NtMPK4       1 MEAISGDQGVQS-NFKGVPTHGGRYAQYNVYGNLFEVSKKYVPL-RPVGRGAYGIVCAAM 58

NtMPK4L-1   60 NSETREEVAIKKIGNAFDNVIDAKRTLREIKLLSHMDHENVIAIKDVIRPPQKKNFNDVY 119
NtMPK4L-2   60 NSETREKVAIKKIGNAFDNVIDAKRTLREIKLLSHMDHENVIAIKDVIRPPQKKNFNDVY 119
NtMPK4      59 NSETREEVAIKKIGNAFDNRIDAKRTLREIKLLRHMDHDNVIATKDIIRPPQTENFNDVY 118

NtMPK4L-1  120 IVYELMDTDLHQIIHSNQQLTDEHCRHFLYQVLRGLKYIHSANILHRDLKPSNLLVNAKC 179
NtMPK4L-2  120 IVYELMDTDLHQIIHSNQQLTDEHCRHFLYQVLRGLKYIHSANILHRDLKPSNLLVNAKC 179
NtMPK4     119 IVYELMDTDLHQIIRSNQQLTDDHCRYFLYQILRGLKYIHSANVLHRDLKPSNLFLNANC 178

NtMPK4L-1  180 DLKIGDFGLARTTTETDFMMEYCVTRWYRAPELLLNCSEYTSAIDVWSVGCILGEILTRQ 239
NtMPK4L-2  180 DLKIGDFGLARTTTETDFMMEYCVTRWYRAPELLLNCSEYTSAIDVWSVGCILGEILTRQ 239
NtMPK4     179 DLKVGDFGLARTTSETDFMTEYVVTRWYRAPELLLNCSEYTAAIDIWSVGCILGEMMTRQ 238

NtMPK4L-1  240 PLFPGRDYVHQLRLITELIGSPDDASLGFLRSNNARRYVRQLPRYPRQQFSARFPNSSPR 299
NtMPK4L-2  240 PLFPGRDYVHQLRLITELIGSPDDASLGFLRSNNARRYVRQLPRYPRQQFSARFPNSSPR 299
NtMPK4     239 PLFPGKDYVHQLKLITELIGSPDDASLGFLRSDNARRYVRQLPQYPRQQFAARFPNSSPG 298

NtMPK4L-1  300 AVDLLEKMLIFDPSRRITVDEALSHPYLAPLHDINEEPVCPRPFSLDFEQPSFTEDNIKE 359
NtMPK4L-2  300 AVDLLEKMLIFDPSRRITVDEALSHPYLAPLHDINEEPVCPRPFSLDFEQPSFTEDNIKE 359
NtMPK4     299 AVDLLEKMLVFDPSRRVTVDQALCHPYLAPLHDINEEPICPKPFSFDFEQPSFTEENIKE 358

NtMPK4L-1  360 LIWREAVKFNPDPTH                                              374
NtMPK4L-2  360 LIWREAVKFNPDPTQ                                              374
NtMPK4     359 LIWRESVKFNPDPTH                                              373
```

Supplementary Figure S3

```
SlMPK5    1 M--EASTGDHGVQSNFRGVPTHGGRYVQYNVYGNLFEVSKKYVPL-RPVGRGAYGIVCAA  57
SlMPK6    1 MSLDSSSADHGGHSNIRGVPTHGGRYVQYNVHGSLFEVSRKYVPPIRPIGRGANGMVCAA  60

SlMPK5   58 LNSETREEVAIKKIGNAFDNRIDAKRTLREIKLLRHMDHDNVIAIRDIIRPPQTENFNDV 117
SlMPK6   61 VNSETREEVAIKKIGNAFDNVIDAKRTLREIKLLSHMDHENVIAIKDVIRPPQKKNFNDV 120

SlMPK5  118 YIVYELMDTDLHQIVRSNQQLTDDHCRYFLYQILRGLKYIHSANVLHRDIKPSNLFLNAN 177
SlMPK6  121 YIVYELMDTDLHQIIHSNQQLTDEHCRHFLYQILRGLKYIHSANILHRDLKPSNLLVNAK 180

SlMPK5  178 CDLKVGDFGLARTTSETDFMTEYVVTRWYRAPELLLNCSEYTAAIDIWSVGCILGEMMTR 237
SlMPK6  181 CDLKIGDFGLARTTTETDFMMEYCVTRWYRAPELLLNCSEYTSAIDIWSVGCILGEILTR 240

SlMPK5  238 QPLFPGRDYVHQLKLITELIGSPDDASLGFLRSDNARRYVRQLPQYPRQQFAAKFPNASP 297
SlMPK6  241 QPLFPGRDYVHQLRLITELIGSPDDASLGFLRSNNARRYVRQLPRYPRQQFAARFPNSSP 300

SlMPK5  298 GAVDLLEKMLVFDPSKRVTVDEALCHPYLAPLHDINEEPVCPRPFSFDFEQPSFTEENIK 357
SlMPK6  301 RAVDLLEKMLIFDPSRRITADEALCHPYLAPLHEINEEPVCPRPFSLDFEQPSLTEDNIK 360

SlMPK5  358 ELIWRETVNFNPDSTH                                             373
SlMPK6  361 ELIWREAAKFNPDPTH                                             376
```

Supplementary Figure S4

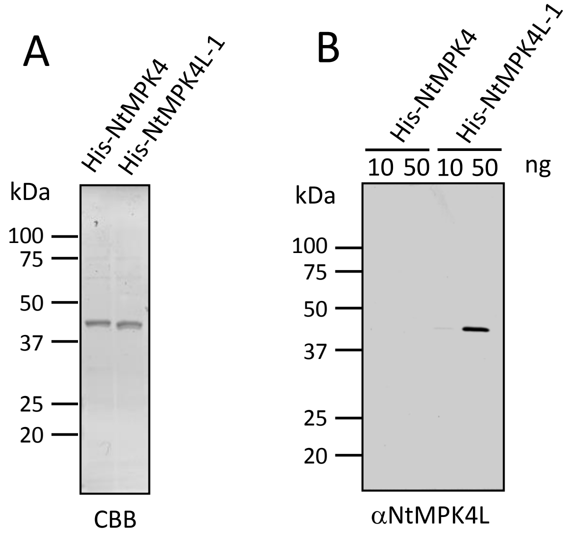

Supplementary Figure S5

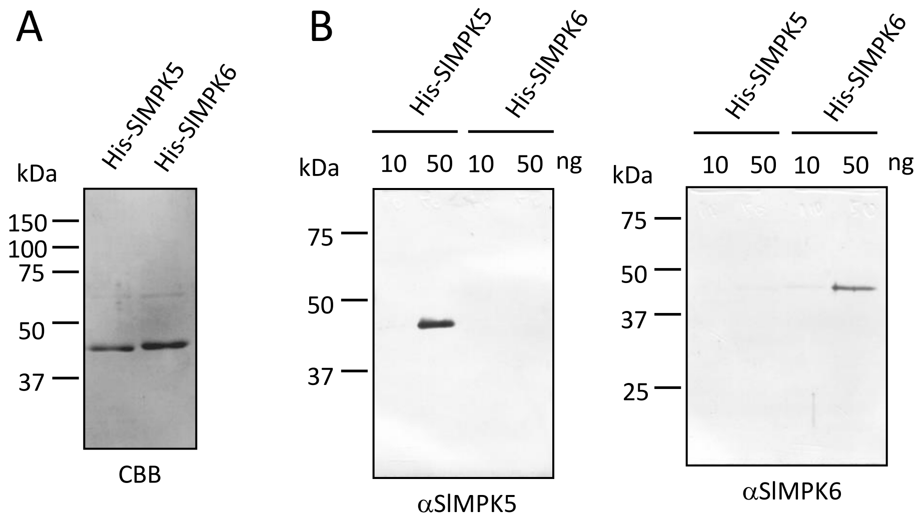

Supplementary Figure S6

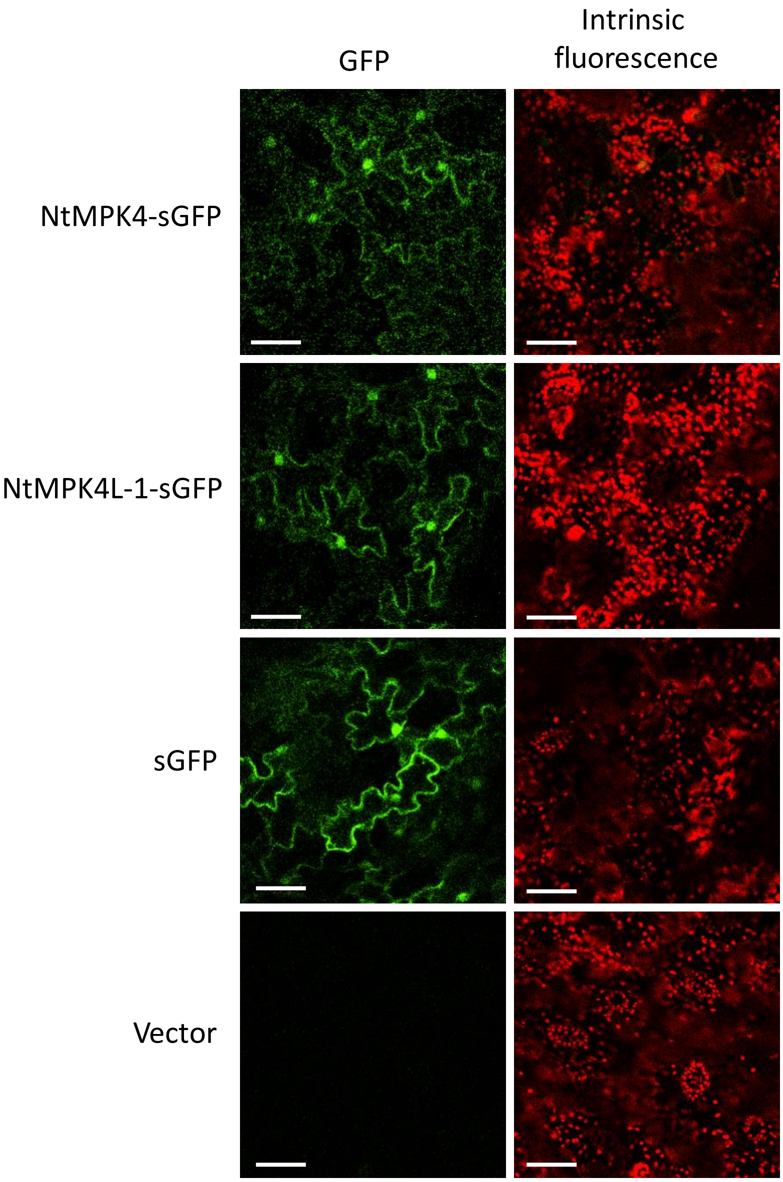

Supplementary Figure S7

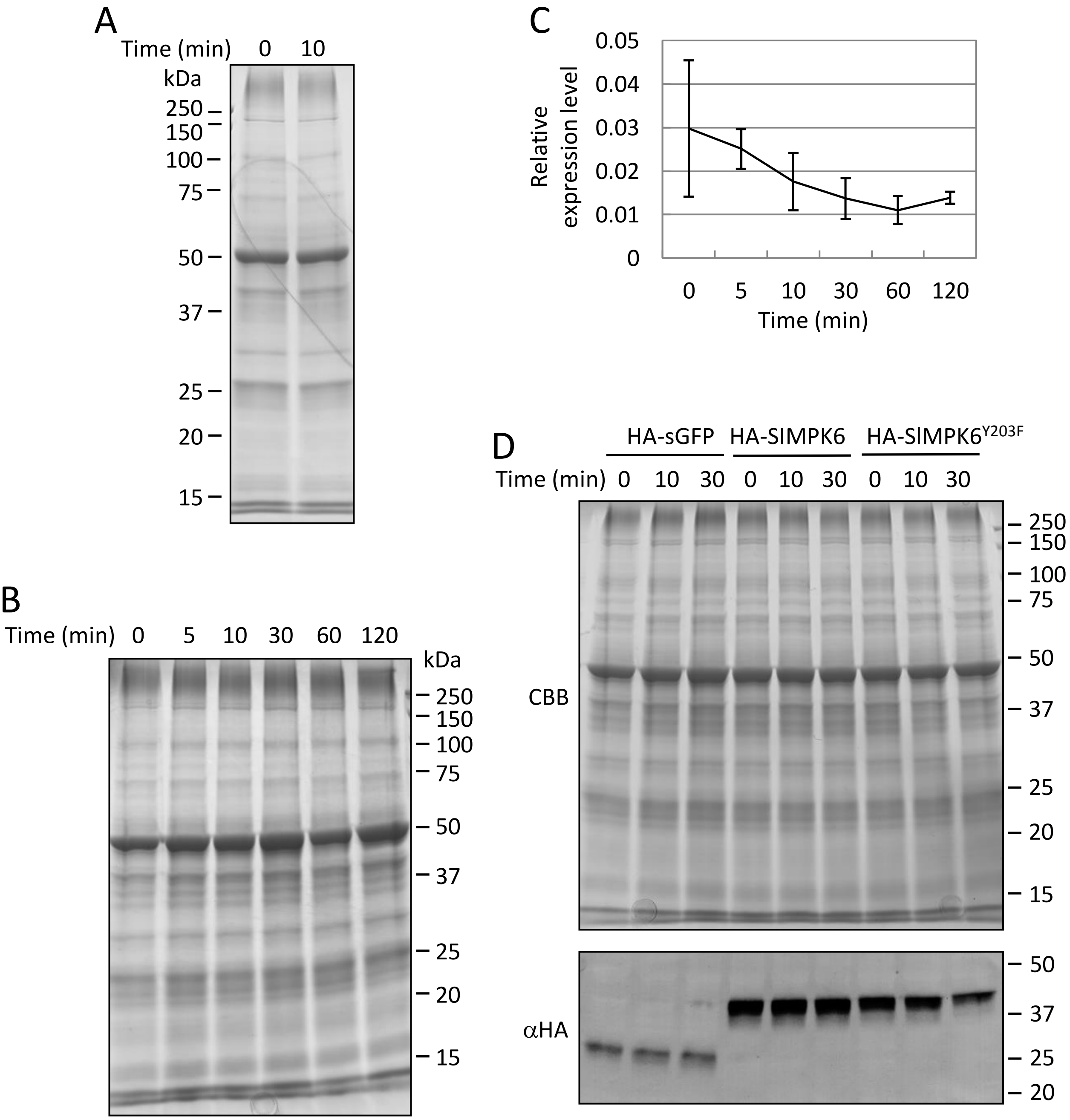

Supplementary Figure S8

A
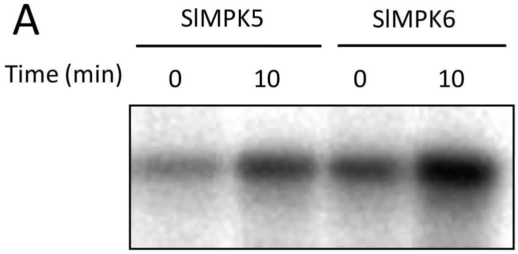

B
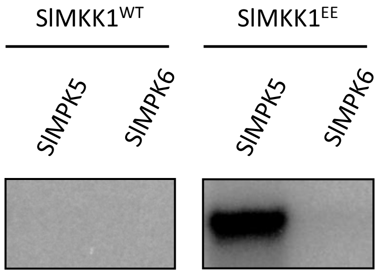

Supplementary Figure S9

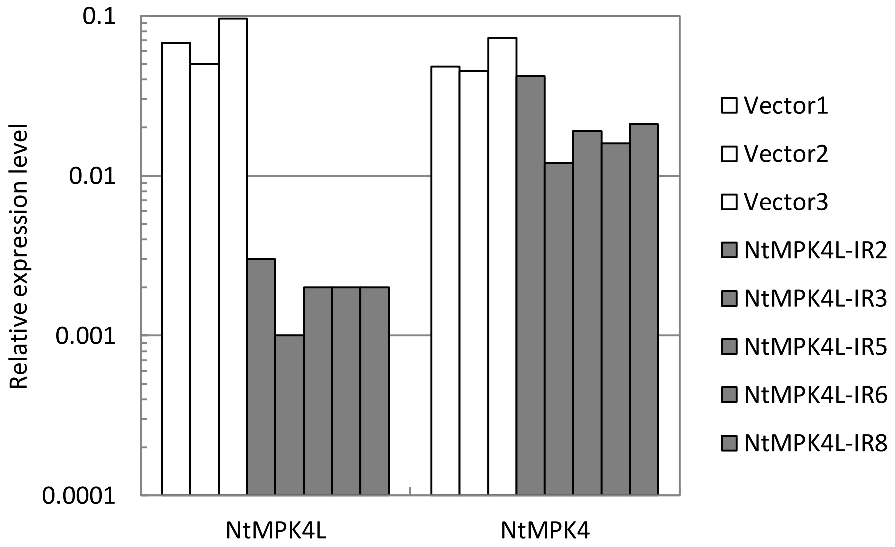

Supplementary Figure S10

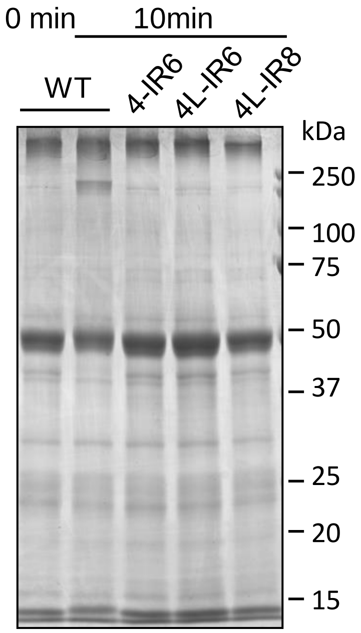

Supplementary Figure S11

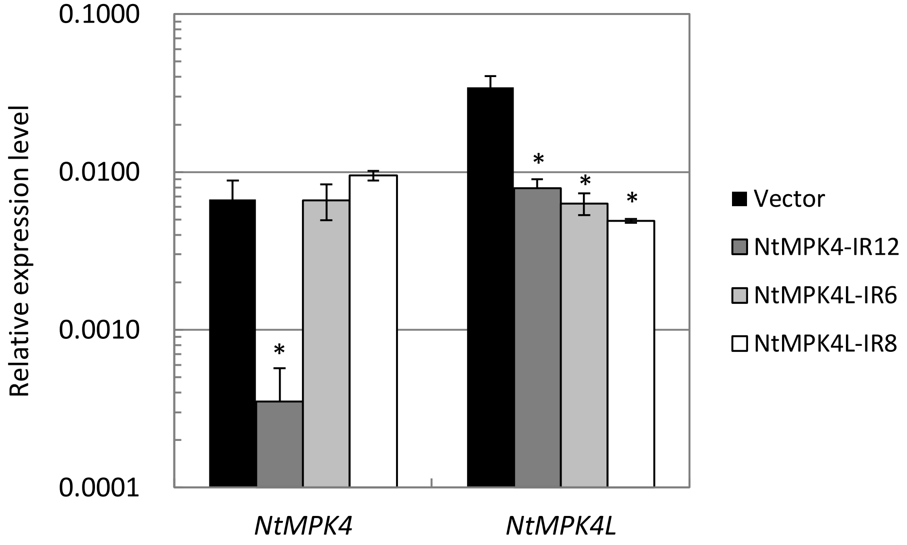

Supplementary Figure S12

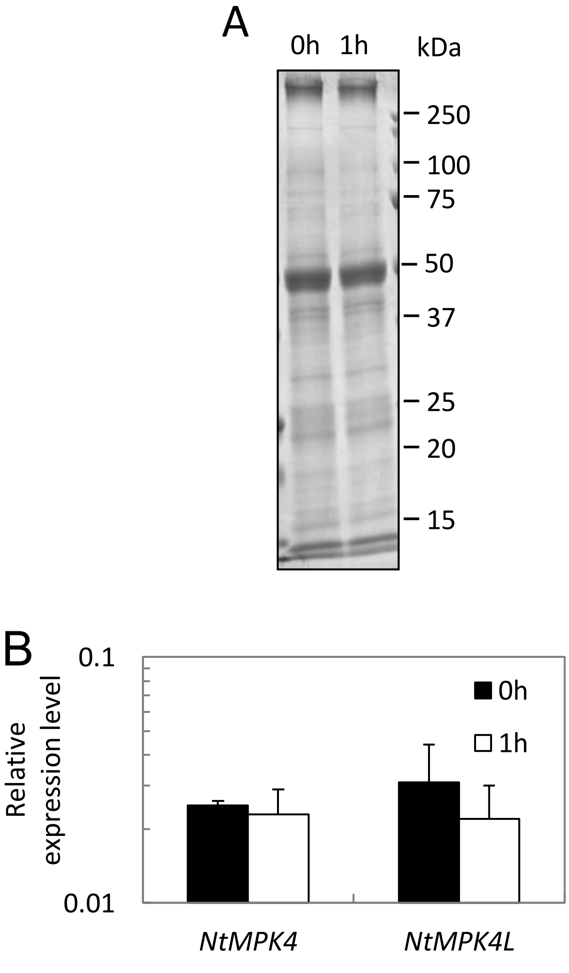

Supplementary Table S1 Primers used in this study

Primer	Sequence (5' – 3')	Application
NtMMPK4L Dege 5'	GGIGTICCIACICAYGGIGG	Cloning
NtMMPK4L Dege 3'	TGIGTIGGRTCIGGRTTRAAYTTIACIGC	Cloning
NtMPK4L 5'	CTGAAATGTCCAAAGGCTCTTC	Cloning
NtMPK4L 3'	GAACTCAACAGCTCCACCATA	Cloning
5'+3'UTR BamHI 5'	TTAGGATCCCTGAAACCAAAGGCTC	Silencing vector
5'UTR+3'UTR TR Rv	CAGGATTAAATTTTACAGCCCCATGTGTTGGTACTCCTC	Silencing vector
3'UTR Fw	GCTGTAAAATTTAATCCTGATCC	Silencing vector
5'+3'UTR XhoI 3'	TTACTCGAGGAACTCAACAGCTCCACC	Silencing vector
5'+3'UTR SacI 5'	TTAGAGCTCCTGAAATGTCCAAAGGCTC	Silencing vector
5'+3'UTR KpnI 3'	TTAGGTACCGAACTCAACAGCTCCACC	Silencing vector
NtActin RT 51	GGGTTTGCTGGAGATGATGCT	qPCR
NtActin RT 31	GCTTCGTCACCAACATATGCAT	qPCR
NtMPK4L-Fw-2	TCAGAGTATACATCGGCAATTGATG	qPCR
NtMPK4L-Rv-2	ATAAGTCTCAACTGGTGTACATAATCTC	qPCR
NtMPK4-5'-1	GTTGGGTTGATTTCCTATGGAAG	qPCR
NtMPK4-rv-1	CATAAACAATGTAGACATCATTGAAATTCTC	qPCR
NtMPK4 NdeI 5'	TTCCATATGATGGAAGCAATTTCAGGTGATC	Recombinant protein
NtMPK4 BamHI 3'	TTCGGATCCTCAGTGAGTTGGATCTGG	Recombinant protein
NtMPK4L NdeI 5'	TTCCATATGATGTCTGTTGATTCGAGTTC	Recombinant protein
NtMPK4L BamHI 3'	TTCGGATCCTCAATGAGTTGGATCAGG	Recombinant protein
5' NdeI-SlMPK5	CTACATATGGATCATGACAATGTGATT	Recombinant protein
3' BamHI-SlMPK5	ATCGGATCCTCAGTGAGTTGAATCTGGA	Recombinant protein
5' NdeI-SlMPK6	CATATGTCTCTTGATTCAAGTTCAGCT	Recombinant protein
3' BamHI-SlMPK6	GGATCCTCAATGAGTTGGATCA	Recombinant protein
5' EcoRI-SlMKK1	GAATTCATGAAGAAAGGATCTTTTGCA	Recombinant protein
3' SalI-SlMKK1	GTCGACTTATAGCTCAGTAAGTGT	Recombinant protein
SlMKK1^{EE} Fw	GCAAGCGAATCTGGACTGGCCAATGAATTTGTC	Recombinant protein
SlMKK1^{EE} Rv	GACAAATTCATTGGCCAGTCCAGATTCGCTTGC	Recombinant protein
SlMPK6YF Fw	TTCATGATGGAATTTTGTGTGACACGCTGGTACCGT	Immuno complex kinase assay
SlMPK6YF Rv	ACGGTACCAGCGTGTCACACAAAATTCCATCATGAA	Immuno complex kinase assay
SlMPK6-BamHI Fw	CTAGGATCCATGTCTCTTGATTCAAGTTCA	Immuno complex kinase assay
SlMPK6-XhoI Rv	ATTCTCGAGTCAATGAGTTGGATCAGGATT	Immuno complex kinase assay
EcoRI-sGFP	TACGAATTCATGGTGAGCAAGGGCGAGG	Immuno complex kinase assay
sGFP-XhoI	AGTCTCGAGTTACTTGTACAGCTCGTCCATGC	Immuno complex kinase assay
SpeI-sGPF-F	TACTAGTATGGTGAGCAAGGGCGAGGAG	Subcellular localization
SacI-sGFP-R	TGAGCTCTTACTTGTACAGCTCGTCCATG	Subcellular localization
XbaI-NtMPK4-F	TATCTAGAATGGAAGCAATTTCAGGTGATCAAG	Subcellular localization
EcoRI-NtMPK4-R	AGAATTCGTGAGTTGGATCTGGATTAAATTTCAC	Subcellular localization
XbaI-NtMPK4L-1-F	TATCTAGAATGTCTGTTGATTCGAGTTCAGG	Subcellular localization
EcoRI-NtMPK4L-1-R	AGAATTCATGAGTTGGATCAGGATTAAATTTTACAGC	Subcellular localization
